# A Novel Biological Activity of Praziquantel Requiring Voltage-Operated Ca^2+^ Channel β Subunits: Subversion of Flatworm Regenerative Polarity

**DOI:** 10.1371/journal.pntd.0000464

**Published:** 2009-06-23

**Authors:** Taisaku Nogi, Dan Zhang, John D. Chan, Jonathan S. Marchant

**Affiliations:** Department of Pharmacology and The Stem Cell Institute, University of Minnesota Medical School, Minnesota, United States of America; Swiss Tropical Institute, Switzerland

## Abstract

**Background:**

Approximately 200 million people worldwide harbour parasitic flatworm infections that cause schistosomiasis. A single drug—praziquantel (PZQ)—has served as the mainstay pharmacotherapy for schistosome infections since the 1980s. However, the relevant *in vivo* target(s) of praziquantel remain undefined.

**Methods and Findings:**

Here, we provide fresh perspective on the molecular basis of praziquantel efficacy *in vivo* consequent to the discovery of a remarkable action of PZQ on regeneration in a species of free-living flatworm (*Dugesia japonica*). Specifically, PZQ caused a robust (100% penetrance) and complete duplication of the entire anterior-posterior axis during flatworm regeneration to yield two-headed organisms with duplicated, integrated central nervous and organ systems. Exploiting this phenotype as a readout for proteins impacting praziquantel efficacy, we demonstrate that PZQ-evoked bipolarity was selectively ablated by *in vivo* RNAi of voltage-operated calcium channel (VOCC) β subunits, but not by knockdown of a VOCC α subunit. At higher doses of PZQ, knockdown of VOCC β subunits also conferred resistance to PZQ in lethality assays.

**Conclusions:**

This study identifies a new biological activity of the antischistosomal drug praziquantel on regenerative polarity in a species of free-living flatworm. Ablation of the bipolar regenerative phenotype evoked by PZQ via *in vivo* RNAi of VOCC β subunits provides the first genetic evidence implicating a molecular target crucial for *in vivo* PZQ activity and supports the ‘VOCC hypothesis’ of PZQ efficacy. Further, in terms of regenerative biology and Ca^2+^ signaling, these data highlight a novel role for voltage-operated Ca^2+^ entry in regulating *in vivo* stem cell differentiation and regenerative patterning.

## Author Summary

Praziquantel is the major drug used to treat people infected with parasitic worms that cause the neglected tropical disease schistosomiasis. Despite being in widespread clinical use, it is surprising that scientists have not identified how praziquantel works to kill pathogenic schistosomes. This lack of pathobiological insight is a major roadblock to the directed design of new drugs to treat schistosomiasis, as the relevant *in vivo* target molecule/pathway of praziquantel remains undefined. In this report, we have discovered a new biological activity of praziquantel that enables us to bring a unique chemical genetic perspective to the problem of identifying molecules needed for *in vivo* praziquantel efficacy. Specifically, we show that praziquantel miscues regenerative patterning in a species of free-living flatworm to yield bipolar (two-headed) organisms. By using this phenotype to screen for molecules underpinning this activity, we provide *in vivo* support for the ‘Ca^2+^ channel hypothesis’ of PZQ efficacy, and show that manipulation of specific subunits of voltage-gated Ca^2+^ channels prevent this effect, and lessen praziquantel-mediated toxicity. These data provide further impetus to studying the role of these proteins in schistosome pharmacotherapy.

## Introduction

Flatworms (‘platyhelminths’) comprise a diverse grouping of ∼25,000 species representing some of the simplest organisms that are triploblastic and bilaterally symmetric. The majority of flatworms are parasitic (tapeworms, flukes and skin/gill ectoparasites) and several are associated with infections in humans and farmed livestock or fish. The most clinically important of these is Schistosomiasis (Bilharzia) caused by infection with trematode flukes of the *Schistosoma* genus that infects ∼200 million people worldwide [1],[2]. With a high morbidity rate associated with chronic infection, it remains one of the most burdensome tropical diseases. Praziquantel (PZQ) has remained the drug of choice for treating Schistosomiasis (and other cestode infections) for over 30 years and remains the focus of country-wide treatment regimens. As the mainstay of pharmacotherapy, the fact that the relevant *in vivo* targets of PZQ remain to be identified prevents rational design of the next generation of antischistosomal chemotherapeutics and is clearly a precarious scenario relative to the potential emergence of drug resistance [3],[4]. A variety of hypotheses have been advanced concerning possible target(s) that mediate PZQ toxicity in schistosomes, encompassing effects on nucleoside uptake [5], phosphoinositide metabolism [6], actin [7], myosin light chain [8], inhibition of glutathione S-transferase [9], and stimulation of Ca^2+^ entry through voltage-operated Ca^2+^ channels (VOCCs, [10],[11]). However, no single target has received unequivocal experimental support, and the relevant *in vivo* molecule(s)/pathway(s) targeted by PZQ remain elusive [2],[5].

A smaller grouping of flatworms (∼10% of species) are free-living planarians (‘turbellarians’). These organisms have a long history of experimental usage owing to their developmental plasticity and remarkable regenerative abilities. For example, small fragments excised from a planarian have the ability to reform a complete body plan [12]–[14]. This ability is driven by a totipotent population of stem cells, called ‘neoblasts’ that populate the planarian mesenchyme. If a cut fragment contains neoblasts, these cells will migrate toward the wounds and replace appropriate cell types from a regenerative structure (‘blastema’) formed at the site(s) of injury. For example, if a trunk fragment is cut from an intact worm, a new ‘head’ will regenerate at the anterior blastema, a ‘tail’ will regenerate from the posterior blastema and other structures will differentiate in a position-dependent manner, thereby reestablishing the anterior-posterior (AP) polarity of the original body plan. Understanding the cellular signaling events which regulate *in vivo* neoblast differentiation to form the <30 planarian cell types in a robust, positionally correct manner has proved to be a problem that has fascinated biologists for almost 200 years [15].

The utility of planarians as a simple model for studying regenerative biology has stimulated optimization of experimental methods, including *in vivo* RNAi [16],[17], to investigate molecular events involved in neoblast maintenance and differentiation. This experimental tractability to *in vivo* RNAi, coupled with our discovery of a simple and striking phenotype elicited by PZQ – anteriorization of regeneration to yield two-headed worms – provided opportunity to bring a fresh perspective to the problem of resolving molecules relevant to *in vivo* PZQ activity. Here, we demonstrate the ability of exogenous PZQ to produce bipolar organisms was (i) phenocopied by modulators of calcium (Ca^2+^) homeostasis, (ii) enhanced by a variety of depolarizing stimuli that activate VOCCs and was (iii) selectively ablated by *in vivo* RNAi of VOCC β subunits [18],[19], but not by a VOCC α subunit. In lethality assays, at higher PZQ doses and exposure intervals, similar resistance to PZQ was induced in worms where VOCC β were targeted for knockdown by RNAi. Consequently, in terms of the long standing problem of identifying biological target(s) of PZQ, these data provide the first *in vivo* genetic support for the ‘Ca^2+^ hypothesis’ of PZQ efficacy [10],[11], albeit from a free-living flatworm species. Furthermore, this novel activity associated with PZQ establishes voltage-operated Ca^2+^ influx as a regulator of stem cell differentiation and patterning of the anterior-posterior axis during flatworm regeneration.

## Materials and Methods

### Planarian husbandry and regenerative assays

An asexual clonal GI strain (Gifu, Iruma river) of *Dugesia japonica* was used in this study [20]. This strain exhibits a robust growth rate (1,000-fold colony expansion over 3 years) and broad drug responsiveness [20]–[22]. Planaria were maintained (∼10,000 worms in 15 L of water in 4 containers) at room temperature (20–23°C) and fed strained chicken liver puree (∼10 ml) once a week. Regenerative assays were performed using 5 day-starved worms (n>20) in pH-buffered artificial water at 22°C (1×Montjuïch salts: 1.6 mM NaCl, 1.0 mM CaCl_2_, 1.0 mM MgSO_4_, 0.1 mM MgCl_2_, 0.1 mM KCl, 1.2 mM NaHCO_3_, pH 7.0 buffered with 1.5 mM HEPES). Drugs were sourced as detailed in Table S1. Isoquinolinone derivatives (Figure S1) were obtained from ChemBridge (San Diego, CA). Phenotypes were scored and archived using a Leica MZ16F stereomicroscope and a QiCAM 12-bit cooled color CCD camera. Drug effects were examined using paired t-tests, with differences considered significant at *P*<0.05. ^45^Ca^2+^ uptake assays (∼53 mCi/ml, PerkinElmer) were performed using trunk fragments incubated in the absence or presence of PZQ (70 µM) for 24 hr and incorporated radioactivity determined after filtration (GF/C, Whatman) by liquid scintillation counting [23].

### *In situ* hybridization

Whole-mount *in situ* hybridization was performed at 55°C in hybridization solution (50% formamide, 5×SSC, 100 µg/ml yeast tRNA, 100 µg/ml heparin sodium salt, 0.1% Tween-20, 10 mM DTT, 5% dextran sulfate sodium salt) incorporating digoxygenin (DIG)-labeled antisense riboprobe (40 ng/ml) denatured at 72°C for 15 min prior to use [20]. A standard mixture of BCIP/NBT in chromogenic reaction solution was used for color development, followed by paraformaldehyde fixation. DIG-labeled antisense riboprobe was synthesized by RNA polymerase (Roche) from using either linearized cDNA plasmid or a PCR fragment as the template. Probe regions, and accession numbers for related gene products, were as follows: *PC2* (1–2285 bp); Inx7 (1–1528 bp; AB189256); Opsin (1–475 bp; AJ421264); Myosin heavy chain (4879–5905 bp; AB015484); Inx3 (1–1,810 bp; AB189253); Hox9 (1–1,491 bp; AB049972); ndk (122–1692 bp; AB071948); Ca_v_β1 (51–1,762 bp; FJ483940); Ca_v_β2 (1–2,017 bp; FJ483939).

### Cloning of Ca_v_β subunits

Total RNA was isolated from 20 intact worms using TRIzol and cDNA subsequently synthesized using the SuperScript III First-Strand Synthesis System (Invitrogen). PCR amplification was performed using degenerative primers (forward 5′-AAYMANGAYTGGTGGAT-3′; reverse, 5′-GCYTTYTGCATCATRTC-3′) for VOCC β subunits and products were cloned into pGEM-T Easy vector (Promega) for sequencing. Full length clones were isolated by step-wise screening a cDNA library previously prepared from regenerating fragments of *D. japonica* [20].

### *In vivo* RNAi

Ca_v_β1 (1653 bp), Ca_v_β2 (1421 bp) and Ca_v_α1.1 (780 bp) sequences were amplified using gene specific primers incorporating Kozak sequence and cloned into the IPTG-inducible vector pDONRdT7 [17] using Gateway BP Clonase (Invitrogen). The *Djsix-1* clone (AJ312218) was from [24]. Sequence within a *Xenopus* clone (IMAGE:4406813) with minimal BLAST homology in the planarian genome database was used as a negative control. *In vivo* RNAi was performed as described previously [17],[25] with minor modifications. Worms were fed a mix of chicken liver and bovine red blood cells containing transformed HT115 bacteria induced to express individual dsRNA constructs over several feeding/regeneration cycles (see below). To assess the efficiency of knock-down, quantitative real-time PCR (qPCR) was performed using a ABI 7500 real-time PCR system (Applied Biosystems) and SYBR GreenER qPCR SuperMix Universal (Invitrogen). cDNA (not containing the RNAi targeted sequence) for each gene was cloned into pGEM-Teasy vector (Promega) and used as a template to create gene-specific standard curves for assessing mRNA levels in samples isolated at equivalent regenerative timepoints from different worms. The mRNA levels of specific genes were compared with controls using planarian *β*-actin to normalize RNA input. Primers were: Ca_v_β1: 5′-AGTATTCAGATTACCCGCCTGACAAT-3′, 5′-CACCAAGATGGATTATCACATGAGA-GA-3′; Ca_v_β2: 5′-AGACACATACTGGACAGCTACTCATCCT-3′,5′-AGCTGAGCTTGTAT-CTGTATTTTTGTTG-3′; *β-actin*: 5′-GGTAAT-GAACGATTTAGATGTCCAGAAG-3′, 5′-TCTGCATACGATCAGCAATACCTGGAT-3′; *six-1*: 5′-CATTTAGTACAAGTGCCACCA-ACATCCA-3′, 5′-GTTGGATGTTCGGATTTTGATGAGTTCA-3′. As a further calibration of qPCR results were compared to those from semi-quantitative RT-PCR using the same sample (data not shown).

## Results

### PZQ causes bipolar regeneration

**Figure 1:**
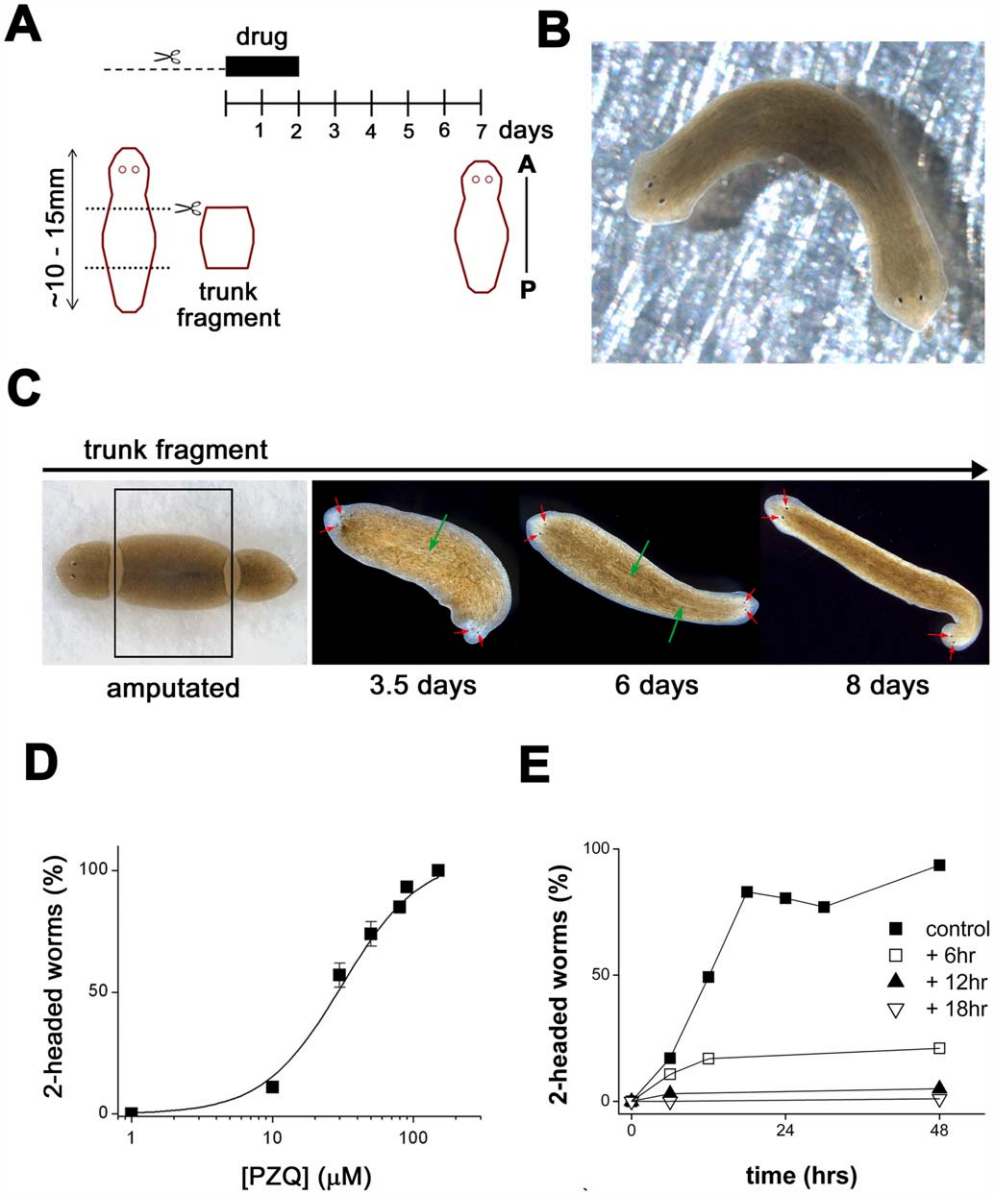
PZQ miscues regeneration to produce two-headed flatworms. (A) Schematic of regenerative assay. Worms were exposed to drugs during trunk fragment isolation (dashed line, mins) and for variable times thereafter (solid bar, days). Anterior-Posterior (AP) polarity was scored at least 7 days after cutting. (B) Two headed flatworm that regenerated from a trunk fragment exposed to PZQ (70 µM, 48 hrs). (C) Regenerative timecourse. Following isolation of trunk fragment (boxed), exposure to PZQ resulted in the appearance of eyespots by 3 to 3.5 days (red arrows), and a second pharynx by 5 to 6 days (green arrows). (D) Dose dependent effect of PZQ on bipolar regeneration (note 100% = all worms regenerated two heads; data is *not* normalized). (E) Duration and timing of PZQ exposure impacted bipolarity. Different plots represent exposure to PZQ (70 µM) offset at different times (square, black triangle, white triangle; 6, 12 & 18 hrs, respectively) after cutting and for the indicated duration (*x*-axis).

Figure 1A depicts a simple, manual screen focused on anterior-posterior (AP) regenerative polarity in the planarian *Dugesia japonica*. In this assay, trunk fragments were cut (heads & tails of worms were amputated) and incubated in drug-containing solution (≤48 hrs), after which the media was exchanged for drug-free solution. Regenerative phenotype was scored after at least 5 days later (i.e. a total of 7 days post-amputation), by quantifying ‘normal’ regeneration (i.e. head structures regenerating at the anterior blastema, and a tail from the posterior blastema) or abnormal phenotypes. The assay was robust and no defects in regenerative polarity were caused by surgery alone in the absence of drug exposure (>1,000 fragments).

Serendipitously, we discovered that exposure to praziquantel (PZQ) in these assays invariably produced two-headed (‘bipolar’) worms from regenerating trunk-fragments (Figure 1B, 87±11% of fragments were bipolar at 70 µM PZQ, n = 5 trials, 285 worms). Bipolarity was first evident after ∼3 to 3.5 days when developing posterior eyespots became apparent and duplicated pharyngeal structures were observed by 5–6 days (Figure 1C). Bipolar worms were viable, able to move, feed and reproduce asexually by splitting (Video S1). This effect of PZQ on regenerative polarity was dose-dependent (EC_50_ = 35±7 µM, Figure 1D) and structure-activity studies confirmed that the ability to evoke bipolarity was retained, albeit with lower potency, by an isoquinoline derivative with high structural similarity to PZQ (Figure S1). However, if PZQ was removed prior to cutting (i.e. samples were washed prior to trunk fragment isolation), bipolarity was not observed. Maximal bipolarity was evoked by exposure to PZQ for only 18 hrs after cutting (Figure 1E). Shifting the time window of PZQ exposure to start 6, 12 or 18 hrs after cutting markedly decreased the anteriorization phenotype (∼50% for 6 hrs, >90% for longer delays, Figure 1E), suggesting that PZQ impacted an early regenerative event. Finally, PZQ also anteriorized regeneration from varied types of fragments cut from varied locations in both asexual and sexualized planarians (Figure S2). There is no prior example of a drug-evoked bipolarity with such robust effectiveness [20], [26]–[31].

### PZQ causes a complete AP axis duplication

**Figure 2:**
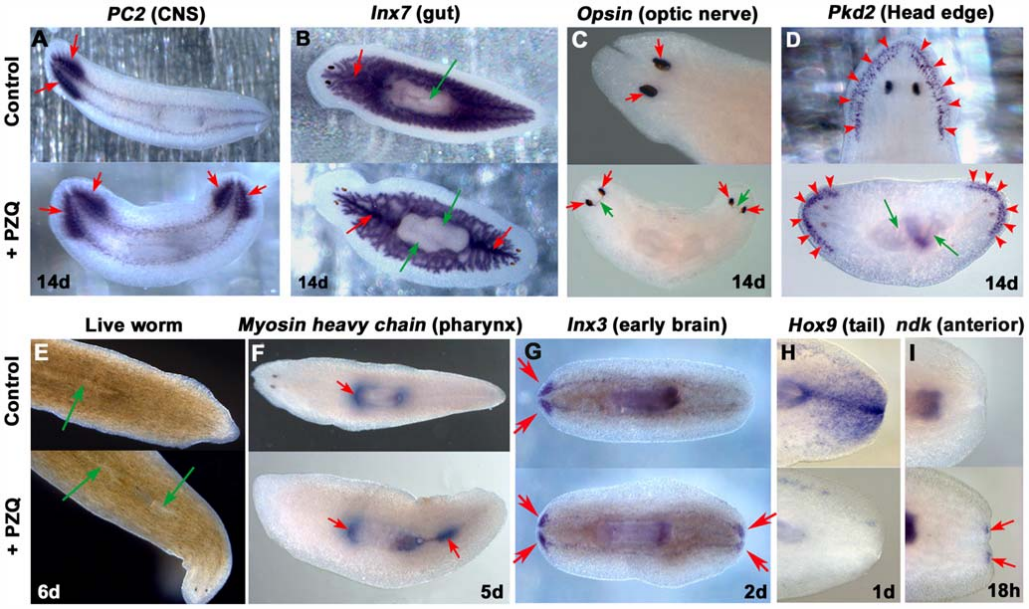
PZQ evokes a complete AP axis duplication. *In situ* hybridization reveals duplication of (A) a CNS marker (prohormone convertase-2 [*PC2*]), (B) a gut marker (innexin-7, [*Inx7*]; red, gut; green, pharynx), (C) an optic nerve marker (*Opsin*; red, photosensors; green, optic nerve), (D) a head edge marker (polycystin-2, [*Pkd2*]), (E) the pharynx (as seen in live worms) and by resolution of the distribution of (F) a pharynx marker (*Myosin*) and (G) an early brain regeneration marker (*Inx3*) in PZQ-treated worms (bottom, 70 µM for 48 hrs) compared to untreated controls (top). (H) A tail enriched marker (*Hox9*) was lost after PZQ treatment. (I) The early anterior marker (ndk) was resolved at the posterior end (and anteriorly) in PZQ-treated worms (bottom) by 18 hrs after cutting compare to untreated controls (top). Worms were fixed 14 days after cutting, with the exception of *ndk* (18 hours), *Hox9* (1 day), *Inx3* (2 days) and *Myosin* (5 days).

How complete was the axis repatterning evoked by PZQ? *In situ* hybridization of tissue-specific mRNAs in PZQ-exposed flatworms demonstrated that PZQ evoked a complete AP axis anteriorization, manifest as a duplication of internal structures along the entire AP axis. CNS markers (prohormone convertase-2 [*PC2*], innexin-3 [*Inx3*]), a gut marker (innexin-7), a head edge marker (polycystin-2 [*Pkd2*]), an optic nerve marker (opsin) and a pharynx marker (myosin) all revealed AP axis duplication in PZQ-treated samples, whereas a tail enriched marker (*Hox9*) was lost within 1 day (Figure 2). Finally, the early brain marker *ndk*, normally localized in the predicted brain region within the anterior blastema, was resolved in the posterior blastema in PZQ-treated samples within 18 hrs post-amputation. These data underscore that PZQ acted early in the regenerative process to dysregulate expression of the earliest known polarity markers (Figure 1E & 2) and that external application of a drug to a living organism induced differentiation of a second set of integrated organs/organ systems, including a dual functional CNS.

### PZQ modulates Ca^2+^ homeostasis

**Figure 3:**
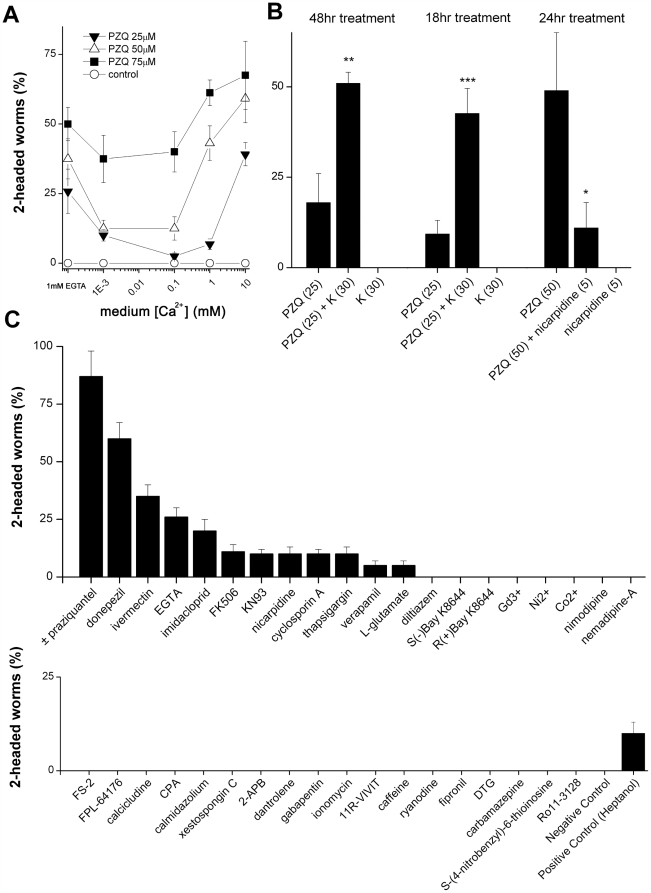
PZQ-evoked bipolarity is Ca^2+^-dependent. (A) Effect of increasing medium Ca^2+^ concentration ([Ca^2+^]_out_: left, Ca^2+^ free (1 mM EGTA); right, 1 µM to 10 mM) on bipolarity evoked by PZQ (24 hr exposure). (B) Left & Middle, [K^+^]_out_ (30 mM) potentiated PZQ-evoked bipolarity (25 µM) over various durations of exposure. Right, nicarpidine (5 µM) antagonized PZQ-evoked bipolarity (50 µM, 24 hrs). Asterisks indicate significance (* = p<5%, ** = p<2%, *** = p<1%) relative to PZQ treatment alone. (C) Screen of Ca^2+^ signaling modulators and selected other agents (48 hr exposure) in the regenerative assay arrayed in terms of decreasing bipolarity. Bipolarity was not observed with the majority of drugs. Drug source, concentration and assay conditions are detailed in Table S1. Positive control is heptanol (350 µM, [20]).

Praziquantel serves as the mainstay pharmacotherapy for schistosomiasis and other cestode infections [2]. Although the relevant *in vivo* molecular target(s) of praziquantel remain undefined [5]–[11], *in vitro* evidence from heterologous expression systems demonstrates that PZQ acts *acutely* to potentiate voltage-operated Ca^2+^ entry [10],[11]. Four pieces of data suggest that PZQ also acts as an activator of Ca^2+^ influx in planarians. First, increased media Ca^2+^ concentrations ([Ca^2+^]_out_) potentiated the efficacy of submaximal concentrations of PZQ at promoting anteriorization (Figure 3A), demonstrating the bipolar phenotype produced by PZQ is facilitated by an increasing gradient for Ca^2+^ entry. Second, net ^45^Ca^2+^ accumulation was higher in regenerating trunk fragments incubated with PZQ compared to regenerating controls (132±13% of controls after 24 hrs, n = 3). Third, although significant increases in [K^+^]_out_ proved toxic, increased [K^+^]_out_ potentiated the ability of submaximal PZQ (25 µM) to produce bipolarity when coincubated with 30 mM K^+^ gluconate (Figure 3B). The maximal extent (∼4.5-fold) of potentiation occurred over an 18 hour period post-cutting (Figure 3B). Finally, nicarpidine, an l-type voltage-operated Ca^2+^ channel antagonist with proven efficacy against voltage-operated Ca^2+^ channels (VOCCs) in flatworms [32], attenuated the ability of PZQ to evoke bipolarity in co-incubation experiments. Bipolarity evoked by 50 µM PZQ decreased by 79±15% in the presence of 5 µM nicarpidine (Figure 3B, n = 3).

However, it was also noted from these assays that manipulations that antagonized Ca^2+^ entry – notably, chelation of media Ca^2+^ with EGTA (Figure 3A&C) or addition of higher concentrations of nicarpidine (>40 µM) in the absence of PZQ (Figure 3C) also produced a small proportion of two-headed flatworms. The penetrance of these phenotypes was much lower (typically ∼10% for pharmacological VOCC inhibition, Figure 3C) than that observed with maximal concentrations of PZQ (∼100% peak effect). The observation that biphasic modulation of Ca^2+^ entry produced bipolarity likely implicates a role for a macroscopic (anterior-posterior) Ca^2+^ gradient in the readout of positional information during regeneration. Such a Ca^2+^ gradient could be flattened by either activating (PZQ) or inhibiting Ca^2+^ entry (see Discussion), but is dampened more effectively by activation (PZQ, ≤100% penetrance) rather than inhibition of a subset of Ca^2+^ entry pathways (∼10% penetrance).

On the basis of these data, we evaluated a broader panel of pharmacological agents known to act as modulators of Ca^2+^ entry and downstream Ca^2+^-dependent effectors in the regenerative assay (Figure 3C, Table S1). Unsurprisingly, the majority of drugs resulted in no regenerative polarity defect: this negative cohort encompasses ‘true’ negatives as well as ‘false’ negatives (drugs that fail to accumulate, or those that lack affinity for invertebrate channels/transporters). However, consistent with the previous data (Figure 3A&B), other depolarizing agents (ivermectin, [33]), or agents that indirectly activate VOCCs (donepezil, imidacloprid, [34]) also evoked bipolarity in a significant proportion of worms (20–60%, Figure 3C). Drugs with low observed incidences of bipolarity (≤10%) comprised inhibitors of molecules involved in Ca^2+^ signaling. In summary, these data show again that either activation (PZQ) or inhibition of Ca^2+^ signaling can miscue regenerative polarity, albeit with different penetrance.

### Characterization of VOCC β subunits in *D. japonica*

On the basis of these results implicating Ca^2+^ homeostasis (Figure 3), we proceeded to investigate the role of voltage-operated Ca^2+^ channels (VOCCs), and notably Ca_v_β subunits in PZQ efficacy [10],[11], using a chemical genetic *in vivo* RNAi approach [17]. Degenerate PCR revealed the presence of two VOCC β subunits in *D. japonica*, from which we proceeded to clone full length sequences (Ca_v_β1, 551 amino acids, ∼62 kDa; Ca_v_β2, 652 amino acids, ∼74 kDa; GENBANK Accession Numbers FJ483939/40). Both these Ca_v_β subunits displayed conservation of key β core domains (SH3, HOOK, guanylate kinase), regulatory motifs and residues crucial for α subunit interaction defined from vertebrate Ca_v_β crystal structures (Figure S3, [18],[19],[35]) and the subunits exhibited ∼47% (Ca_v_β1) and ∼33% (Ca_v_β2) overall identity to human CACNB1/2 (Table S2). *D. japonica* Ca_v_β1 displayed ∼57% sequence identity to *Schistosoma mansoni* (*Sm*) Ca_v_β, while sequence identity was lower between Ca_v_β2 and *Sm* Ca_v_β_var_ (34%, Table S2). *In situ* hybridization revealed widespread distribution of β subunit mRNA in mesenchyme, brain (Ca_v_β1 & Ca_v_β2) and pharyngeal muscle (Ca_v_β1>Ca_v_β2, Figure S3C). Similarly, RT.PCR screening for individual Ca_v_β subunits in different cut sections of worms (head, trunk and tail) confirmed mRNA for both Ca_v_β subunits was present in trunk fragments used in the regenerative assay (Figure S3D).

**Figure 4:**
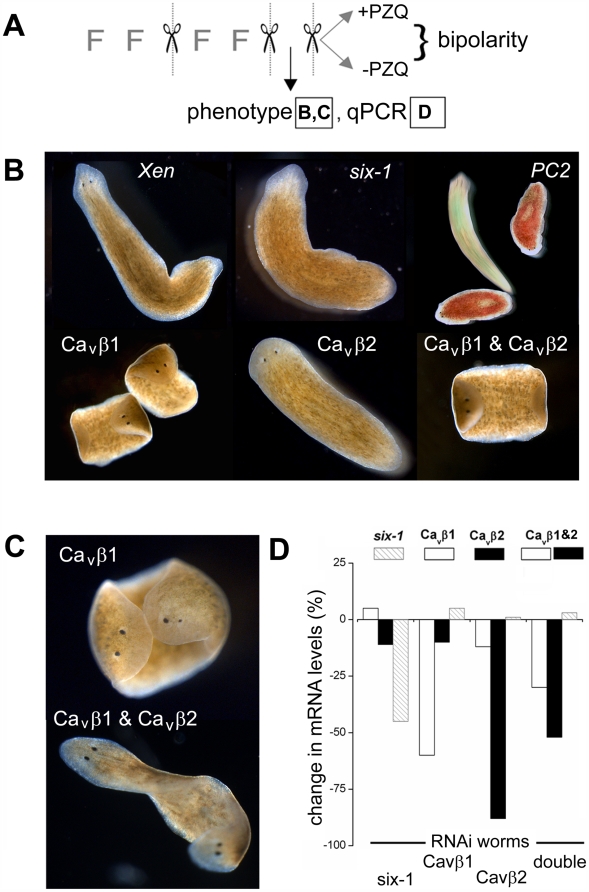
RNAi of Ca_v_β subunits in *D. japonica*. (A) Schematic overview of protocol for *in vivo* RNAi assays. Phenotype of worms was scored after two cycles of feeding (≤2 feedings) and cutting. At this point, a sample of worms (≤10 worms) were used for qPCR, and the remaining worms were split equally into two cohorts to assay regeneration in the presence or absence of PZQ. Regenerative phenotypes are shown in (B & C) and qPCR data in (D). (B) Images of RNAi phenotypes using indicated constructs after two regenerative cycles. For the *PC2* panel, worms were stained with food coloring to identify control (green) and *PC2* RNAi-treated cohorts (red) and a snapshot taken over a long exposure to highlight the difference in mobility between these groups. (C) Bipolar planarian produced via Ca_v_β1 (top), or dual Ca_v_β1 and Ca_v_β2 knockdown (bottom) in the absence of PZQ exposure. (D) qPCR of abundance of indicated mRNAs(legend at top) in worms fed indicated dsRNA constructs (bottom). ‘Double’ represents co-feeding with Ca_v_β1 and Ca_v_β2.

To investigate the *in vivo* role of each Ca_v_β subunit, worms were fed bacteria expressing dsRNA against Ca_v_β1 or Ca_v_β2, as well as constructs serving as positive (*six-1*, a transcription factor essential for to eye regeneration [24]; *PC2*, a enzyme needed for photoaversion [36]) and negative RNAi controls (*Xen*, see Materials and Methods) over multiple feeding/regenerative cycles (Figure 4A). After the second regenerative cycle, phenotypic effects were recorded (Figure 4B&C) and compared to results from real time PCR analysis from the same cohort of worms to quantify the effectiveness and specificity of *in vivo* RNAi (Figure 4D). Finally, a third regenerative cycle was performed during which half the worms were exposed to PZQ (with the other half remaining untreated) for the purpose of assaying whether knockdown of specific mRNAs impacted the ability of PZQ to evoke bipolarity. In total, from first feeding to final phenotypic scoring, each independent assay was ∼1 month in duration.

In this assay, we observed no phenotypic effect in worms fed the negative control (*Xen*) construct (n = 4 independent cycles, as per Figure 4A). As expected, worms fed the positive control (*six-1*) construct failed to develop eyespots after regeneration (∼90% of worms lacked eyespots after the second regenerative cycle, Figure 4B). Similarly, after the second regenerative cycle, worms subject to *PC2* RNAi display impaired mobility (Figure 4B, Figure S4). *In vivo* RNAi of Ca_v_β1 and Ca_v_β2 subunits yielded distinct phenotypic outcomes. Knockdown of Ca_v_β1 disrupted worm motility and feeding, leading to first ‘corkscrewing’ (Video S2) and then ‘curled’ immobilized worms (Figure 4B, Video S3). These defects in motility, observed in Ca_v_β1 and *PC2* RNAi worms, did not however prevent regeneration. Knockdown of Ca_v_β2 did not produce any motility defect (Video S4). The most apparent phenotype in Ca_v_β2 RNAi worms was a rounded (as opposed to normally ‘arrowed’) head morphology (Figure 4B). Finally, in double VOCC β subunit knockdown worms (Ca_v_β1&2), both the mobility and morphological phenotypes were apparent.

Importantly, *in vivo* RNAi of VOCC β subunits modulated AP polarity. Knockdown of Ca_v_β1, either alone or in combination with Ca_v_β2, yielded a small proportion of bipolar worms in the absence of PZQ exposure (4.8±2.8% in Ca_v_β1; 8.0±4.0% in Ca_v_β1 and Ca_v_β2, n = 4; Figure 4C). The magnitude of this effect was similar to the small proportion of bipolar worms resulting from pharmacological blockage of VOCCs (∼10%, Figure 3C), confirming that either genetic or pharmacological inhibition of voltage-operated Ca^2+^ entry miscued regenerative polarity, albeit with a low peak effect by either approach.

To determine the specificity and effectiveness of *in vivo* RNAi, we performed real time PCR analysis (Figure 4D). Quantification by real-time PCR revealed effective knockdown of the targeted mRNAs, ranging from a decrease of ∼40% (*six-1*) to ∼90% (Ca_v_β2) in single knockdowns. The double knockdown (Ca_v_β1 & Ca_v_β2) was also effective, although the magnitude of knockdown of individual β subunits was lower compared to the levels achieved in worms fed either construct alone. Finally, knockdown was selective: there was no significant change in β subunit mRNA in worms fed a control construct (*six-1*) and despite the identity (∼37%) between the two VOCC β subunits, Ca_v_β1/2 mRNA levels changed <15% in the reciprocal (Ca_v_β2/1) single β subunit knockdown (Figure 4D).

### PZQ-evoked bipolarity is ablated by RNAi of VOCC β subunits

**Figure 5:**
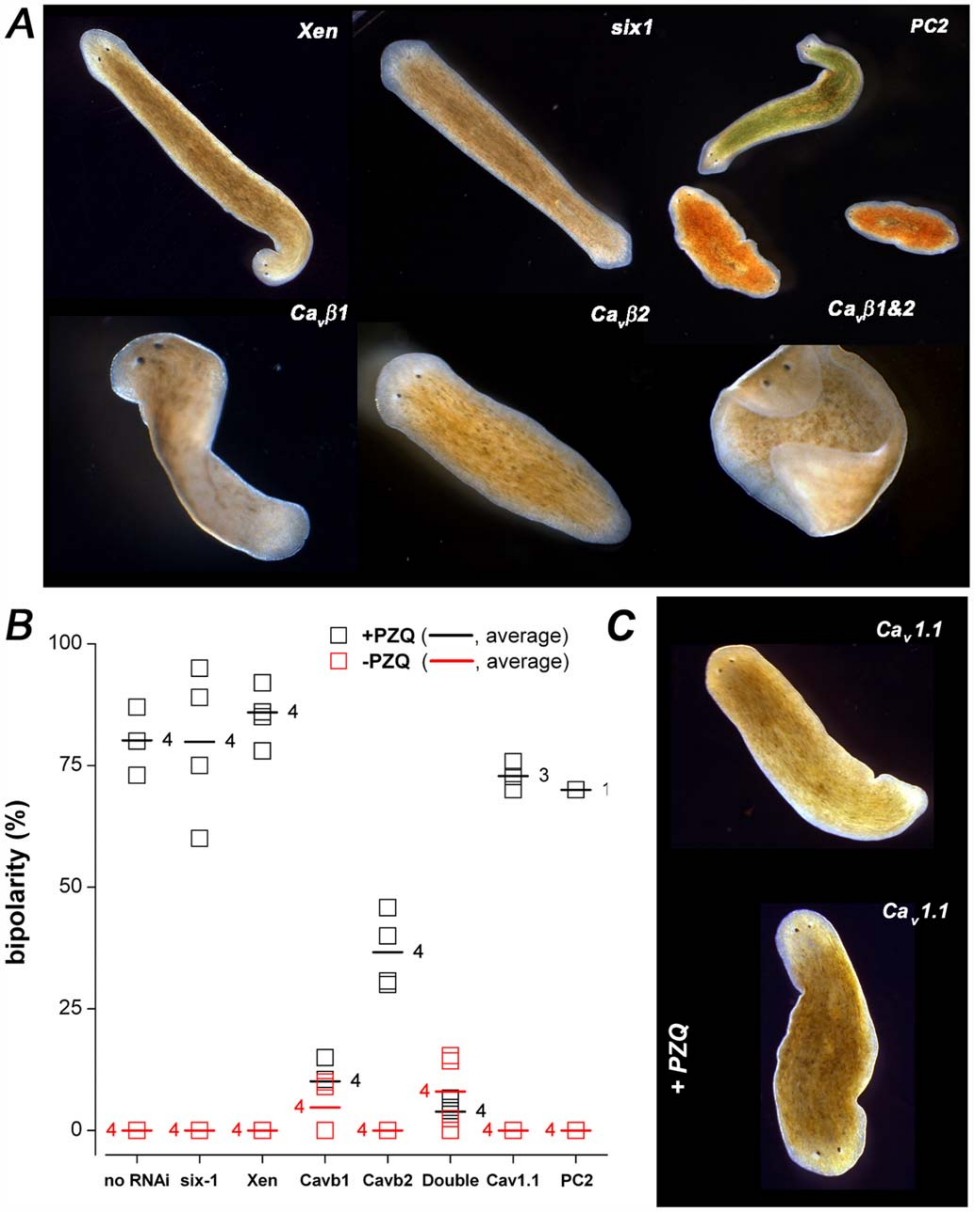
Ca_v_β RNAi ablated PZQ-evoked bipolarity. (A) Images of phenotypes produced by PZQ exposure (70 µM for 24 hrs) in worms fed the indicated dsRNA constructs (after a third regenerative cycles). (B) Scoring of bipolarity in different cohorts of dsRNA-fed worms exposed to PZQ (black squares) or left untreated (red squares) through a third regenerative cycle. Each square represents the percentage of bipolar worms (not normalized) in a single assay. The horizontal line represents the average of the indicated number of experiments for each construct. The number of independent experiments for each cohort is indicated. (C) Representative images of Ca_v_1.1 RNAi worms in the absence (top) or presence of PZQ (70 µM for 24 hrs, bottom).

To test the effectiveness of PZQ in evoking bipolarity in the different RNAi cohorts following manipulation of β subunit levels, trunk fragments were cut and allowed to regenerate either in the presence or absence of PZQ. In both the negative and positive RNAi controls, PZQ (70 µM, 24 hrs) induced two-headed (*Xen*) or two-headed, no-eyed worms (*six-1*) worms with similar effectiveness to that observed in the naïve ‘no-RNAi’ cohort (Figure 5A). No bipolarity was observed in *six-1* or *Xen* worms (>100 trunk fragments) in the absence of PZQ exposure (Figure 5B). Similarly, immobilization of worms via *PC2* RNAi failed to prevent PZQ-evoked bipolarity, and genetic bipolarity was not observed in this cohort in the absence of PZQ exposure (Figure 5A&B).

In contrast, Ca_v_β1 knockdown (alone or in combination with Ca_v_β2) antagonized the ability of PZQ to produce bipolar worms. Only a small percentage of bipolar worms were evoked by PZQ in Ca_v_β1 (10.1±3.5%) and double (Ca_v_β1 and Ca_v_β2, 3.9±1.4%) knockdown worms, and this residual number was similar to the percentage of ‘genetic’ two-heads (Figure 4C). These results are consistent with earlier pharmacological and physiological evidence (Figure 2) that PZQ stimulates Ca^2+^ entry through VOCCs, as this activity was ablated by knockdown of VOCC subunits. Knockdown of Ca_v_β2 also attenuated PZQ evoked-bipolarity (Figure 5B) but the extent of the inhibition was less pronounced than with Ca_v_β1 (36.7±3.8% vs 10.1±3.5% bipolarity for Ca_v_β2 or Ca_v_β1 knockdown, respectively), despite a near complete loss of Ca_v_β2 mRNA (Figure 4D). These data suggest that Ca_v_β2 is a less effective mediator of PZQ-evoked bipolarity, although we cannot exclude the possibility that Ca_v_β2 is irrelevant to PZQ-evoked bipolarity and the partial attenuation simply results from the small decrease in Ca_v_β1 levels (∼12%) seen in Ca_v_β2 worms (Figure 4D). Finally, to assess whether PZQ-evoked bipolarity could be prevented by knockdown of other Ca^2+^ channels subunits, we developed a further RNAi construct (see Methods) targeting one of the several pore-forming VOCC α subunits expressed in *D. japonica* (Ca_v_1.1, Zhang *et al.* unpublished data). In worms treated with this constructs, PZQ was still effective at evoking bipolarity (Figure 5B&C). Therefore, the ability of VOCC β subunit ablation to attenuate PZQ-evoked bipolarity was specific to the manipulation of certain VOCC subunits, and notably Ca_v_β1. Conservatively, these *in vivo* RNAi data collectively demonstrate that knockdown of VOCC β subunits attenuated PZQ-evoked bipolarity.

### Ca_v_β RNAi provides resistance to PZQ toxicity

**Figure 6:**
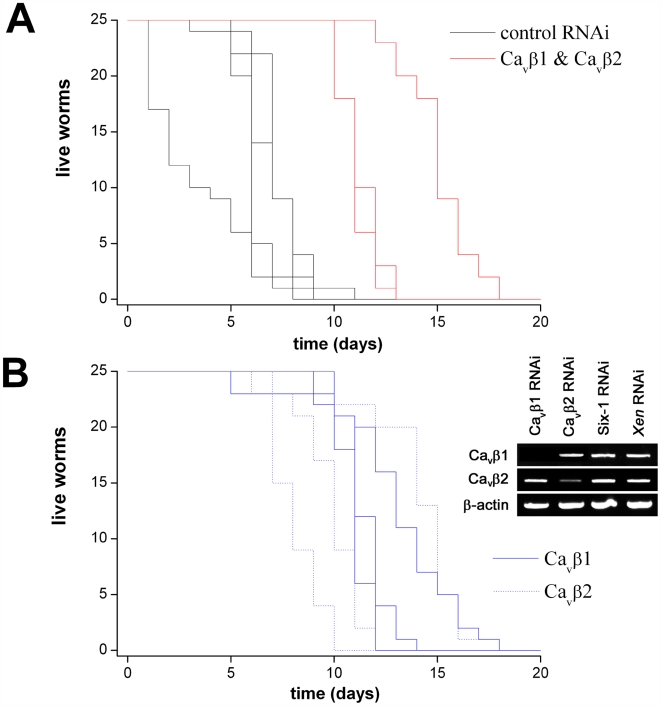
PZQ-evoked lethality in *D. japonica*. (A) Intact worm survival over time in two cohorts of worms fed either a control (black, *Xen*) or dual Ca_v_β (red, Ca_v_β1 & Ca_v_β2 knockdown) RNAi vectors. Each line represents a survival curve in an independent assay (n = 3). (B) Survival plot in worms where individual Ca_v_β subunits were targeted by RNAi. *Inset*, RT.PCR data from Cavβ and control cohorts (top) to assess changes in Ca_v_β1 & Ca_v_β2 mRNA abundance.

Finally, we tested whether *in vivo* RNAi of Ca_v_β subunits provided resistance to PZQ-evoked lethality in *intact* worms. First, we performed toxicity testing to evaluate the concentration range of maintained exposure to PZQ that resulted in lethality in *D. japonica*. Figure 6 shows that *intact* worms, subjected to multiple feeding cycles with the negative (‘Xen’) RNAi control, began to die within a few days when continually incubated in 100 µM PZQ (LD_50_ = 7.0±2.4 days, n = 4). In parallel assays, the double Ca_v_β1 and Ca_v_β2 RNAi cohort exhibited heightened resistance to PZQ exposure, surviving for almost twice as long (LD_50_ = 13.3±2.3 days, n = 3). Analysis of survival curves in single knockdown cohorts (i.e. Ca_v_β1 or Ca_v_β2), resolved lethality over a timeframe between the control and double knockdown (LD_50_ = 12.7±1.1 for Ca_v_β1, 11.7±3.1 for Ca_v_β2 respectively, n = 3). RT.PCR analysis of intact worms fed individual Ca_v_β constructs confirmed knockdown was selective in the cohort of worms used for this survival time assay (Fig. 6B). Therefore, these data demonstrate that manipulation of Ca_v_β levels also afforded protection against PZQ toxicity in intact worms.

## Discussion

Here, we report a novel biological activity of the antischistosomal praziquantel by illustrating an unexpected activity of this drug, and close structural mimetics, to anteriorize regeneration of fragments cut from a species of free-living flatworm. By chemical genetic analysis we show that PZQ-evoked bipolarity, as well as PZQ-evoked toxicity, was attenuated by *in vivo* RNAi of VOCC β subunits. While such data do not identify VOCC β subunits as the direct target of PZQ –only as gene products epistatic to PZQ action – when considered in conjunction with previous whole cell current analysis of VOCC properties in the presence of heterologously overexpressed schistosome β subunits [10],[11],[37], they add further, and crucially, *in vivo* genetic support for the ‘Ca^2+^ channel hypothesis’ of PZQ action. Therefore, we believe these results bring a fresh, albeit unorthodox, perspective to the problem of defining molecules crucial for PZQ efficacy *in vivo*. By taking a phylogenetic side-step to a different class of platyhelminths, relevance to schistosomal physiology is certainly an extrapolation: however, we note that Ca_v_β subunits are well conserved across between different species (trematodes, cestodes as well as turbellarians) that show sensitivity to PZQ [37].

A couple of differences between these data and the previous heterologous expression experiments in *Xenopus* oocytes deserve comment [10],[11]. Prior molecular evidence demonstrated that PZQ acted to potentiate Ca^2+^ entry through heterologously expressed non-flatworm VOCCs (<2-fold change in peak current) contingent on overexpression of a ‘variant’ (Ca_v_β_var_, [10],[11],[37]) schistosome VOCC β subunit. This ‘variant’ β subunit lacks two consensus PKC phosphorylation sites shown by site-directed mutagenesis as being crucial for conferring PZQ sensitivity, such that when consensus phosphorylation motifs are reintroduced, PZQ sensitivity is lost [10],[11],[37]. Although *D. japonica* Ca_v_β2 also lacks the consensus PKC phosphorylation sites defined as being critical for conferring PZQ sensitivity in heterologous systems (Figure S3), in our experiments PZQ efficacy was more critically dependent on Ca_v_β1 levels (where the consensus PKC phosphorylation sites are present). A second difference relates to timescale: the heterologous current recordings reveal an *acute* action of PZQ (secs), while our *in vivo* data reveal an effect that is manifest chronically (Figure 1E, 18 hrs for peak exposure effect). It is interesting to note that PZQ, originally developed in a synthesis of novel anti-anxiety compounds, shares some structural similarity to benzodiazepines, several of which upregulate the expression of specific VOCC subtypes in neurons [38]. Therefore, both acute (potentiation of existing currents) as well as chronic effects of PZQ (upregulation of VOCC expression) merit further examination.

**Figure 7:**
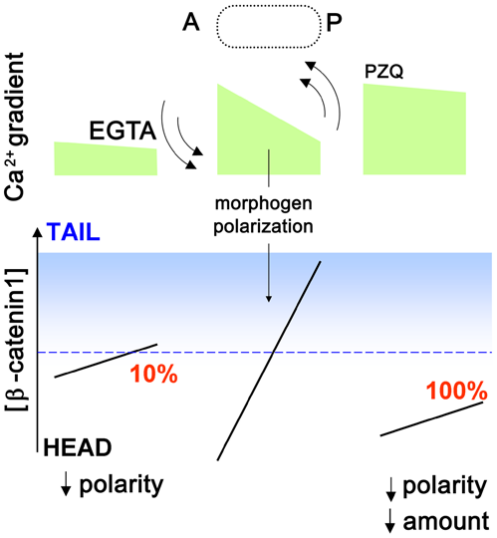
Possible role of a Ca^2+^ gradient in regulating AP polarity of *D. japonica*. A speculative model to address how either activation (PZQ) or inhibition (EGTA) of Ca^2+^ influx can evoke bipolarity albeit with different penetrances (100% vs 10%). The key tenets of the model are (i) a critical level of β-catenin-1 (blue) is needed to specify ‘tail’ fate [43], (ii) a macroscopic Ca^2+^ gradient in regenerating fragments (green) directly/indirectly polarizes AP β-catenin-1 distribution and (iii) high Ca^2+^ attenuates canonical Wnt signaling to decrease β-catenin-1 levels [40],[43]. *Right*, PZQ activates Ca^2+^ influx to flatten the distribution *and* decrease β-catenin-1 levels, resulting in a consistent anteriorization of regenerative blastemas (100% penetrance). *Left*, Inhibition of Ca^2+^ influx by EGTA, VOCC inhibitors, or Ca_v_β RNAi treatment decreases the polarization of existing morphogen, making posterior fate decisions less reliable (∼10% penetrance).

A second area of significance of these studies relates to regenerative patterning. As a remarkably penetrant effect on the polarity of regeneration in *D. japonica*, these data provide novel impetus to define the epigenetic role played by Ca^2+^ signals in regulating *in vivo* stem cell differentiation and regenerative specification, expanding the versatility of differently sourced Ca^2+^ signals in regulating the patterning of different body axes in different organisms [39]–[41]. Particularly curious is the observation from both the RNAi and pharmacological screens demonstrating that AP polarity is miscued by both activation and inhibition of VOCCs, albeit far more effectively by activating (PZQ) than inhibiting Ca^2+^ influx. One speculative model for AP fate consistent with the experimental data is shown in Figure 7. It has been demonstrated recently that blastema polarity within the excised trunk fragment is determined by β-catenin-1 [25],[42], such that tail specification occurs above a critical local (nuclear) threshold of β-catenin-1. If Ca^2+^ regulates both the gradient of positional identity *and* is antagonistic to β-catenin-1 stability [43], then changes in Ca^2+^ influx will impact both the distribution of positional cues as well as the overall concentration of β-catenin-1. Flattening the macroscopic Ca^2+^ gradient by inhibiting Ca^2+^ influx through VOCCs flattens the gradient of positional identity, leading to less robust posterior fate decisions (∼10% misspecification). Praziquantel, by activating Ca^2+^ influx through VOCCs, also flattens the Ca^2+^ gradient controlling positional identity, but crucially also decreases β-catenin-1 levels thereby leading to consistent anteriorization outcomes. The model is consistent with the antagonistic role of Ca^2+^ signals on canonical Wnt signaling established for patterning events in other systems [43]. However, direct validation of this model would require Ca^2+^ imaging experiments, which would be facilitated by future optimization of transgenic methods in this system to enable global expression of genetically-coded Ca^2+^ indicators competent to resolve changes in resting free Ca^2+^ concentration and Ca^2+^ influx during regeneration. Indeed, planaria harbor a broad diversity of neurotransmitters that could regionally stimulate VOCCs during regeneration [44]. Finally, as a safe, clinically approved drug, PZQ treatment provides a cheap and facile method for generating bipolar organisms for laboratory experiments and teaching demonstrations. Planarians are commonly used model organisms in the classroom to showcase the phenomenon of regeneration [45]. Useful for studies spanning molecular regeneration of the CNS [46] to whole animal behavioral responses [47], the ability to induce dual, integrated central nervous systems by exogenous drug application is a striking visual outcome.

## Supporting Information

Table S1 Drug screen of Ca^2+^ homeostasis modulators. For simplicity of presentation, the only heteromorphy scored was full bipolarity (i.e. dual heads). Less complete anteriorization phenotypes (e.g. no tail, duplicated pharynx) were observed with several drugs, but not incorporated into the scoring matrix, likely under representing the efficacy of several compounds in anteriorizing regenerative events. Concentrations for assays were determined after first performing toxicity tests to identify the highest concentration range that did not affect worm viability. Drug exposures were <2 day, and in cohorts of >30 worms for n≥3 independent trials. Positive control is heptanol (350 µM, [20]). Negative control represents surgery in the absence of drug exposure (>1000 fragments). PZQ was used as a racemic mixture (±PZQ).
(0.10 MB DOC)

Table S2 Identity table of VOCC β subunits. Full length polypeptides were aligned using the BLOSUM62 scoring matrix (ClustalW MSA). The following accession identifiers were used: *Schistosoma mansoni* (Sm) Ca_v_β (gi15283999, [10]), Sm Ca_v_β_var_ (gi15283996, [11]), *Homo sapiens* (Hs) CACNB1 (gi20455481) and CACNB2 (gi123238417).
(0.04 MB DOC)

Figure S1 Structure-activity screening of isoquinolinone derivatives. (A) Structure of PZQ. (B) Three compounds were screened for activity (150 µM, 24 hrs): compound ‘a’ (2-benzoyl-1,2,3,6,7,11b-hexahydro-4H-pyrazino[2,1-a]isoquinolin-4-one), compound ‘b’ (7,11b-dihydro-2H-pyrazino[2,1-a]isoquinoline-1,4(3H,6H)-dione), and compound ‘c’ (2-(2-phenylethyl)-7,11b-dihydro-2H-pyrazino[2,1-a]isoquinoline-1,4(3H,6H)-dione). (C) Incidence of bipolarity from a 24 hr incubation with the indicated concentration of each drug (µM).
(0.28 MB DOC)

Figure S2 PZQ is efficacious at anteriorizing different types of planarian fragments. (A) Image and schematic representation of basic trunk fragment assay, where two cuts (dashed) yield three fragments (‘head’, ‘trunk’ and ‘tail’ fragments) and four blastemas (‘1’–‘4’). (B) Results from the procedure shown in (A) scored as proportion of fragments regenerating heads from indicated blastema (‘1’–‘4’) in control (open bars) and PZQ-treated worms (solid bars, 70 µM, 24 hr). Number of fragments shown as italicized label from a cumulative dataset. (C) Gallery of bipolar worms produced by PZQ (70 µM, 24 hr) treatment from different cut fragments (cartoons) from asexual (i–iii, brown cartoon) and sexualized (iv & v, blue cartoon) worms. For each example, proportion of bipolar fragments by PZQ (70 µM, 24 hr) treatment are shown from the total number of cut segments. Examples represent (i) short (1–2 mm) pre- and post-pharyngeal fragments, (ii) anterior (‘A’) blastema cut from trunk fragment exposed to PZQ (70 µM) for 1 day, (iii) posterior (‘P’) blastema cut from trunk fragment exposed to PZQ (70 µM) for 1 day, (iv) head fragment of sexualized *D. japonica* and (v) trunk fragment of sexualized *D. japonica* worm.
(2.52 MB DOC)

Figure S3 Characterization of *D. japonica* Ca_v_β1 and Ca_v_β2. (A) Alignment of *D. japonica* Ca_v_β1 (551 amino acids) and Ca_v_β2 subunits (652 amino acids). Identical residues are shown in yellow, similar residues in green. Both proteins show highest homology in their SH3 (blue) and guanylate kinase domains (red) as illustrated in the similarity projection in (B). Residues in the GK domain shown to wall the α-interacting domain pocket are highlighted (*, [35]).Residues previously implicated in determining PZQ sensitivity are shown (arrow, [10],[11]). (C) *In situ* hybridization of Ca_v_β1 (top) and Ca_v_β2 mRNA (bottom) in ventral view shown in intact worms (left) and regenerating worms (2 days post cutting, right). Staining in pharynx (green arrows) and brain (red arrows is shown). Ca_v_β2 staining occurs in the anterior and posterior of the pharynx region (*). (D) RT.PCR analyses of mRNA distribution in head (‘h’), trunk fragments (‘p’ for pharynx) and tail (‘t’) sections for Ca_v_β subunits, as well as loading controls (β-actin) and regional markers (‘opsin’, head-specific; ‘Hox9’ posteriorly-biased marker). Primers: *actin*: 5′-TGGGACGATATGGAGAAGATCTGGCAT-3′, 5′-GCATACGATCAGCAATACCAGGGTA-CA-3′; *opsin*: 5′-CACCGCCATTTTTTGGTTTGGAAA-3′, 5′-GCAAATAGCACTGGTAGTT-CAGCAG-3′; *hox9*: 5′-GATTCTGCCTTCGGTAAATCTGACAT-3′; 5′-GCAATTTCCCACG-TTTTTGTCTAGT-3′; *Myosin*: 5′-AACGACGAACTGAAATGCCACCTCA-3′; 5′-CAGCTTG-TTCTTCTCTGGGTCTTTGT-3′; Ca_v_β1: 5′-TTACAAGATGCATGTGAACATC-3′; 5′-TAA-ATGGAGAATGCGCTATATC-3′; Ca_v_β2: 5′-ATTCAACAAATAAAATAAAAACTC-3′, 5′-TCGATATCCCAATATTCTATCTGC-3′.
(1.64 MB DOC)

Figure S4 Effect of *PC2* RNAi on worm mobility following sudden light exposure. Intact worms subject to *PC2* RNAi (stained with red food color), and controls (stained green) were placed in a drop of water and video frames captured at 2 second intervals following exposure to white light. Stills show that *PC2* RNAi worms (red) remain relatively immobile relative to the worms exhibiting the light aversion response (green).
(0.64 MB DOC)

Video S1 Two headed planarians evoked by PZQ (120 frames, 40 ms per frame)
(10.23 MB MOV)

Video S2 ‘Corkscrewing’ planarian produced by *in vivo* RNAi of Cav-beta1 (100 frames, 90 ms per frame). Early phenotype (3 days after completion of feeding cycles).
(9.08 MB MOV)

Video S3 Immobilized, ‘curled’ planarians produced by *in vivo* RNAi of Cavbeta1 (100 frames, 90 ms per frame). Late phenotype (1 week after completion of feeding cycles).
(9.99 MB MOV)

Video S4 Representative example showing lack of impaired planarian mobility (100 frames, 90 ms per frame) resulting from *in vivo* RNAi of Cavbeta2 (1 week after completion of feeding cycles).
(4.53 MB MOV)

## References

[1] Ross AGP, Bartley PB, Sleigh AC, Olds GR, Li Y (2002). Schistosomiasis.. NEJM.

[2] Caffrey CR (2007). Chemotherapy of schistosomiasis: present and future.. Curr Opin Chem Biol.

[3] Ismail M, Bortos S, Metwally A, William S, Farghally A (1999). Resistance to praziquantel: direct evidence from Schistosoma mansoni isolated from Egyptian villagers.. Am J Trop Med Hyg.

[4] Fallon PG, Doenhoff MJ (1995). Drug-resistant schistosomiasis: resistance to praziquantel and oxamniquine induced in *Schistosoma mansoni* in mice is drug specific.. Am J Trop Med Hyg.

[5] Angelucci F, Basso A, Bellelli A, Brunori M, Pica Mattoccia L (2007). The anti-schistosomal drug praziquantel is an adenosine antagonist.. Parasitol.

[6] Wiest PM, Li Y, Olds R, Bowen WD (1992). Inhibition of phosphoinositide turnover by praziquantel in *Schistosoma mansoni*.. J Parasitol.

[7] Tallima H, El Ridi R (2007). Praziquantel binds *Schistosoma mansoni* adult worm actin.. Int J Antimicrob Agents.

[8] Gnanasekar M, Salunkhe AM, Mallia AK, He YK, Kalyanasundaram R (2009). Praziquantel affects the regulatory myosin light chain of Schistosoma mansoni.. Antimicrob Agents Chemother.

[9] McTigue MA, Williams DR, Tainer JA (1995). Crystal structures of a schistosomal drug and vaccine target: Glutathione S-transferase from *Schistosoma japonica* and its complex with the leading antischistosomal drug praziquantel.. J Mol Biol.

[10] Kohn AB, Anderson PAV, Roberts-Misterly JM, Greenberg RM (2001). Schistosome calcium channel β subunits.. J Biol Chem.

[11] Kohn AB, Roberts-Misterly JM, Anderson PAV, Khan N, Greenberg RM (2003). Specific sites in the beta interaction domain of a schistosome Ca^2+^ channel β subunit are key to its role in sensitivity to the anti-schistosomal drug praziquantel.. Parasitol.

[12] Morgan TH (1898). Experimental studies of the regeneration of *Planaria maculata*.. Arch Entwm Org.

[13] Newmark PA, Sanchez-Alvarado A (2002). Not your father's planarian: a classic model enters the era of functional genomics.. Nat Rev Genet.

[14] Agata K, Watanabe K (1999). Molecular and cellular aspects of planarian regeneration.. Cell & Dev Biol.

[15] Dalyell JG (1814). Observations on Some Interesting Phenomena in Animal Physiology exhibited by several species of planariae.

[16] Newmark PA, Reddien PW, Cebria F, Sanchez Alvarado A (2003). Ingestion of bacterially expressed double-stranded RNA inhibits gene expression in planarians.. Proc Natl Acad Sci.

[17] Reddien PW, Bermange AL, Murfitt KJ, Jennings JR, Sánchez Alvarado A (2005). Identification of genes needed for regeneration, stem cell function, and tissue homeostasis by systematic gene perturbation in planaria.. Dev Cell.

[18] Hidalgo P, Neely A (2007). Multiplicity of protein interactions and functions of the voltage-gated calcium channel β-subunit.. Cell Calcium.

[19] Dolphin AC (2003). β subunits of voltage-gated calcium channels.. J Bioenerg Biomemr.

[20] Nogi T, Levin M (2005). Characterization of innexin gene expression and functional roles of gap-junctional communication in planarian regeneration.. Dev Biol.

[21] Nogi T, Yuan YE, Sorocco D, Perez-Thomas R, Levin M (2005). Eye regeneration assay reveals an invariant functional left-right asymmetry in the early bilterian, *Dugesia japonica*.. Laterality.

[22] Kitamura Y, Inden M, Sanada H, Takata K, Taniguchi T (2003). Inhibitory effects of antiparkinsonian drugs and caspase inhibitors in a parkinsonian flatworm model.. J Pharmacol Sci.

[23] Robinson LC, Marchant JS (2008). Enhanced Ca^2+^ leak from ER Ca^2+^ stores induced by hepatitis C NS5A protein.. BBRC.

[24] Mannini L, Rossi L, Deri P, Gremigni V, Salvetti A (2004). *Djeyes absent* (*Djeya*) controls prototypic planarian eye regeneration by cooperating with the transcription factor *Djsix-1*.. Dev Biol.

[25] Gurley KA, Rink JC, Sánchez-Alvarado A (2008). β-catenin defines head versus tail identity during planarian regeneration and homeostasis.. Science.

[26] Kanatani H (1958). Formation of bipolar heads induced by demecolcine in the planarian, *Dugesia gonocephala*.. J Fac Sci Tokyo Univ.

[27] Flickinger RA (1959). A gradient of protein synthesis in planaria and reversal of axial polarity of regenerates.. Growth.

[28] Rustia CP (1925). The control of biaxial development in the reconstitution of pieces of planaria.. J Exp Zool.

[29] Teshirogi W (1955). The effects of lithium chloride on head-frequency in *Dugesia Gonocephela*.. Bulletin of the Marine Biological Station of Asamushi.

[30] McWhinnie MA (1955). The effect of colchicine on reconstitutional development in *Dugesia dorotocephala*.. Bio Bull.

[31] Rodriguez LV, Flickinger RA (1971). Bipolar head regeneration in planaria induced by chick embryo extracts.. Biol Bull.

[32] Mendonca-Silva DL, Novozhilova E, Cobbett PJR, Silva CLM, Noel F (2006). Role of calcium influx through voltage-operated calcium channels and of calcium mobilization in the physiology of *Schistosoma mansoni* muscle contractions.. Parasitol.

[33] Pérez-Serrano J, Grosman C, Urrea-París MA, Denegri G, Casado N (2001). Depolarization of the tegument precedes morphological alterations in Echinococcus granulosus protoscoleces incubated with ivermectin.. Parasitol Res.

[34] Jepson JEC, Brown LA, Sattelle DB (2006). The actions of the neonicotinoid imidacloprid on cholinergic neurons of Drosophila melanogaster.. Invert Neurosci.

[35] Chen YH, Li MH, Zhang Y, He LL, Yamada Y (2004). Structural basis of the alpha1-beta subunit interaction of voltage-gated Ca^2+^ channels.. Nature.

[36] Kass J, Jacob TC, Kim P, Kaplan JM (2001). The EGL-3 Proprotein Convertase Regulates Mechanosensory Responses of *Caenorhabditis elegans*.. J Neurosci.

[37] Jeziorski MC, Greenberg RM (2006). Voltage-gated calcium channel subunits from platyhelminths: potential role in praziquantel action.. Int J Parasitol.

[38] Katsura M, Shibasaki M, Kurokawa K, Tsujimura A, Ohkuma S (2007). Up-regulation of L-type high voltage-gated calcium channel subunits by sustained exposure to 1,4- and 1,5-benzodiazepines in cerebrocortical neurons.. J Neurochem.

[39] Kume S, Muto A, Inoue T, Suga K, Okano H (1997). Role of the inositol 1,4,5-trisphosphate receptor in ventral signaling in *Xenopus* embryos.. Science.

[40] Saneyoshi T, Kume S, Amasaki Y, Mikoshiba K (2002). The Wnt/calcium pathway activates NF/AT and promotes ventral cell fate in *Xenopus* embryos.. Nature.

[41] Raya A, Kawakami Y, Rodriguez-Esteban C, Ibanes M, Rasskin-Gutman D (2004). Notch activity acts as a sensor for extracellular calcium during vertebrate left-right determination.. Nature.

[42] Petersen CP, Reddien PW (2008). Smed-*βcatenin-1* is required for anteroposterior blastema polarity in planarian regeneration.. Science.

[43] Slusarski DC, Pelegri F (2007). Calcium signaling in vertebrate embryonic patterning and morphogenesis.. Dev Biol.

[44] Ribeiro P, El-Shehabi F, Patocka N (2005). Classical transmitters and their receptors in flatworms.. Parasitol.

[45] Gibbs MA (2003). A practical guide to developmental biology.

[46] Cebrià F (2007). Regenerating the central nervous system: how easy for planarians!. Dev Genes Evol.

[47] Raffa RB, Rawls SM (2008). Planaria: a model for drug action and abuse.

