# Supplementary material for: A Novel Biological Activity of Praziquantel Requiring Voltage-Operated Ca2+ Channel β Subunits: Subversion of Flatworm Regenerative Polarity
Source: PLoS Negl Trop Dis. 2009 Jun 23;3(6):e464. doi: 10.1371/journal.pntd.0000464 (PMC2694594; doi:10.1371/journal.pntd.0000464)
Supplement: Table S1 — Drug screen of Ca2+ homeostasis modulators. For simplicity of presentation, the only heteromorphy scored was full bipolarity (i.e. dual heads). Less complete anteriorization phenotypes (e.g. no tail, duplicated pharynx) were observed with several drugs, but not incorporated into the scoring matrix, likely under representing the efficacy of several compounds in anteriorizing regenerative events. Concentrations for assays were determined after first performing toxicity tests to identify the highest concentration range that did not affect worm viability. Drug exposures were <2 day, and in cohorts of >30 worms for n≥3 independent trials. Positive control is heptanol (350 µM, [20]). Negative control represents surgery in the absence of drug exposure (>1000 fragments). PZQ was used as a racemic mixture (±PZQ). (0.10 MB DOC) [file pntd.0000464.s001.doc]

**Supplementary Table 1. Drug screen of Ca^2+^ homeostasis modulators**

|  | Drug | Source | Conditions | Bipolarity | % |
| --- | --- | --- | --- | --- | --- |
| 1 | **± praziquantel** | Sigma, LKT, Alexis | 70μM, 48 hrs | **Yes** | **87** |
| 2 | **donepezil** | Eisai | 150μM, 48 hrs | **Yes** | **60** |
| 3 | **ivermectin** | Sigma | 1.4μM, 24 hrs | **Yes** | **35** |
| 4 | **EGTA** | Sigma | 1.5mM, 48 hrs | **Yes** | **26** |
| 5 | **imidacloprid** | Sigma | 100mM, 48 hrs | **Yes** | **20** |
| 6 | **FK-506** | Sigma | 5μM, 48 hrs | **Yes** | **11** |
| 7 | **KN-93** | Calbiochem | 10μM, 48 hrs | **Yes** | **10** |
| 8 | **nicarpidine** | Sigma | 40μM, 48 hrs | **Yes** | **10** |
| 9 | **cyclosporin A** | Sigma | 5μM, 48 hrs | **Yes** | **10** |
| 10 | **thapsigargin** | Sigma | 1μM, 48 hrs | **Yes** | **10** |
| 11 | **verapamil** | Sigma | 20μM, 48 hrs | **Yes** | **5** |
| 12 | **L-glutamate** | Sigma | 1mM, 48 hrs | **Yes** | **5** |
| 13 | diltiazem | Sigma | 40μM, 48 hrs | No | 0 |
| 14 | S(-)Bay K8644 | Sigma | 10μM, 48 hrs | No | 0 |
| 15 | R(+)Bay K8644 | Sigma | 10μM, 48 hrs | No | 0 |
| 16 | Gd^3+^ | Sigma | 50μM, 48 hrs | No | 0 |
| 17 | Ni^2+^ | Sigma | 100μM, 48 hrs | No | 0 |
| 18 | Co^2+^ | Sigma | 1mM, 48 hrs | No | 0 |
| 19 | nimodipine | Alomone Labs | 500μM, 48 hrs | No | 0 |
| 20 | nemadipine-A | Sigma | 5μM, 48 hrs | No | 0 |
| 21 | FS-2 | Calbiochem | 5μM, 48 hrs | No | 0 |
| 22 | FPL-64176 | Sigma | 10μM, 48 hrs | No | 0 |
| 23 | calcicludine | Sigma | 100μM, 48 hrs | No | 0 |
| 24 | CPA | Sigma | 20μM, 48 hrs | No | 0 |
| 25 | calmidazolium | Calbiochem | 50μM, 48 hrs | No | 0 |
| 26 | xestospongin C | Sigma | 1μM, 48 hrs | No | 0 |
| 27 | 2-APB | Sigma | 1μM, 48 hrs | No | 0 |
| 28 | dantrolene | Sigma | 100μM, 48 hrs | No | 0 |
| 29 | gabapentin | Sigma | 4.5mM, 48 hrs | No | 0 |
| 30 | ionomycin | Sigma | 1μM, 48 hrs | No | 0 |
| 31 | 11R-VIVIT | Calbiochem | 5μM, 48 hrs | No | 0 |
| 32 | caffeine | Sigma | 5mM, 48 hrs | No | 0 |
| 33 | ryanodine | Sigma | 1mM, 48 hrs | No | 0 |
| 34 | fipronil | Sigma | 100μM, 48 hrs | No | 0 |
| 35 | DTG | Sigma | 230μM, 48 hrs | No | 0 |
| 36 | carbamazepine | Sigma | 500μM, 48 hrs | No | 0 |
| 37 | *S-(4-nitrobenzyl)-6-thioinosine* | Sigma | 100μM, 48 hrs | No | 0 |
| 38 | *Ro11-3128* | Roche | 50μM, 48 hrs | No | 0 |
| 39 | Negative Control | n/a | n/a | No | 0 |
| 40 | Positive Control (**Heptanol**) | Sigma | 350μM, 48 hrs | **Yes** | **10** |

**Supplementary Table 1**

For simplicity of presentation, the only heteromorphy scored was full bipolarity (i.e. dual heads). Less complete anteriorization phenotypes (e.g. no tail, duplicated pharynx) were observed with several drugs, but not incorporated into the scoring matrix, likely under representing the efficacy of several compounds in anteriorizing regenerative events. Concentrations for assays were determined after first performing toxicity tests to identify the highest concentration range that did not affect worm viability. Drug exposures were <2 day, and in cohorts of >30 worms for n≥3 independent trials. Positive control is heptanol (350μM, [20]). Negative control represents surgery in the absence of drug exposure (>1000 fragments). PZQ was used as a racemic mixture (±PZQ).
