# Supplementary material for: A Novel Biological Activity of Praziquantel Requiring Voltage-Operated Ca2+ Channel β Subunits: Subversion of Flatworm Regenerative Polarity
Source: PLoS Negl Trop Dis. 2009 Jun 23;3(6):e464. doi: 10.1371/journal.pntd.0000464 (PMC2694594; doi:10.1371/journal.pntd.0000464)
Supplement: Table S2 — Identity table of VOCC β subunits. Full length polypeptides were aligned using the BLOSUM62 scoring matrix (ClustalW MSA). The following accession identifiers were used: Schistosoma mansoni (Sm) Cavβ (gi15283999, [10]), Sm Cavβvar (gi15283996, [11]), Homo sapiens (Hs) CACNB1 (gi20455481) and CACNB2 (gi123238417). (0.04 MB DOC) [file pntd.0000464.s002.doc]

**Supplementary Table 2. Identity table of VOCC β subunits.**

|  | *Dj*  **Ca_v_β1** | *Sm *  **Ca_v_β_var_** | *Sm *  **Ca_v_β** | *Hs*  **CACNB1** | *Hs*  **CACNB2** |
| --- | --- | --- | --- | --- | --- |
| *Dj*  **Ca_v_β2** | 37 % | 34 % | 34 % | 33 % | 32 % |
| *Dj*  **Ca_v_β1** |  | 38 % | 57 % | 47 % | 47 % |
| *Sm *  **Ca_v_β_var_** |  |  | 36% | 35 % | 34 % |
| *Sm *  **Ca_v_β** |  |  |  | 41 % | 43 % |
| *Hs*  **CACNB1** |  |  |  |  | 63 % |

Full length polypeptides were aligned using the BLOSUM62 scoring matrix (ClustalW MSA). The following accession identifiers were used: *Schistosoma mansoni* (Sm) Ca_v_β (gi15283999, [10]), Sm Ca_v_β_var_ (gi15283996, [11]), *Homo sapiens* (Hs) CACNB1 (gi20455481) and CACNB2 (gi123238417).
