# Supplementary material for: A Novel Biological Activity of Praziquantel Requiring Voltage-Operated Ca2+ Channel β Subunits: Subversion of Flatworm Regenerative Polarity
Source: PLoS Negl Trop Dis. 2009 Jun 23;3(6):e464. doi: 10.1371/journal.pntd.0000464 (PMC2694594; doi:10.1371/journal.pntd.0000464)
Supplement: Figure S1 — Structure-activity screening of isoquinolinone derivatives. (A) Structure of PZQ. (B) Three compounds were screened for activity (150 µM, 24 hrs): compound ‘a’ (2-benzoyl-1,2,3,6,7,11b-hexahydro-4H-pyrazino[2,1-a]isoquinolin-4-one), compound ‘b’ (7,11b-dihydro-2H-pyrazino[2,1-a]isoquinoline-1,4(3H,6H)-dione), and compound ‘c’ (2-(2-phenylethyl)-7,11b-dihydro-2H-pyrazino[2,1-a]isoquinoline-1,4(3H,6H)-dione). (C) Incidence of bipolarity from a 24 hr incubation with the indicated concentration of each drug (µM). (0.28 MB DOC) [file pntd.0000464.s003.doc]

**Supplementary Figure 1**

**
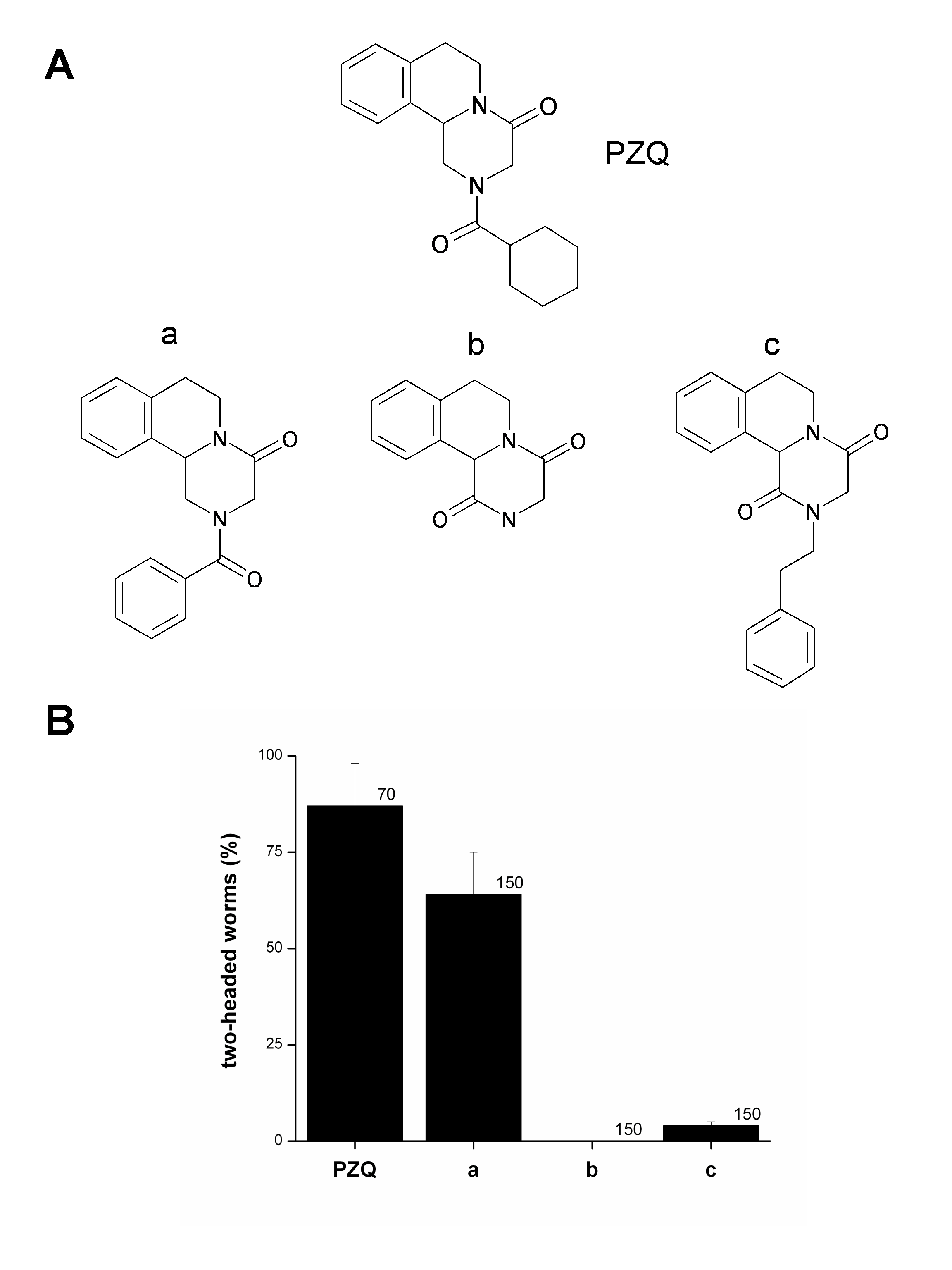
**

**Supplementary Figure 1**

**Structure-activity screening of isoquinolinone derivatives.** (**A**) Structure of PZQ. (**B**) Three compounds were screened for activity (150μM, 24hrs): compound ‘a’ (2-benzoyl-1,2,3,6,7,11b-hexahydro-4H-pyrazino[2,1-a]isoquinolin-4-one), compound ‘b’ (7,11b-dihydro-2H-pyrazino[2,1-a]isoquinoline-1,4(3H,6H)-dione), and compound ‘c’ (2-(2-phenylethyl)-7,11b-dihydro-2H-pyrazino[2,1-a]isoquinoline-1,4(3H,6H)-dione). (C) Incidence of bipolarity from a 24hr incubation with the indicated concentration of each drug (μM).
