# Supplementary material for: A Novel Biological Activity of Praziquantel Requiring Voltage-Operated Ca2+ Channel β Subunits: Subversion of Flatworm Regenerative Polarity
Source: PLoS Negl Trop Dis. 2009 Jun 23;3(6):e464. doi: 10.1371/journal.pntd.0000464 (PMC2694594; doi:10.1371/journal.pntd.0000464)
Supplement: Figure S2 — PZQ is efficacious at anteriorizing different types of planarian fragments. (A) Image and schematic representation of basic trunk fragment assay, where two cuts (dashed) yield three fragments (‘head’, ‘trunk’ and ‘tail’ fragments) and four blastemas (‘1’–‘4’). (B) Results from the procedure shown in (A) scored as proportion of fragments regenerating heads from indicated blastema (‘1’–‘4’) in control (open bars) and PZQ-treated worms (solid bars, 70 µM, 24 hr). Number of fragments shown as italicized label from a cumulative dataset. (C) Gallery of bipolar worms produced by PZQ (70 µM, 24 hr) treatment from different cut fragments (cartoons) from asexual (i–iii, brown cartoon) and sexualized (iv & v, blue cartoon) worms. For each example, proportion of bipolar fragments by PZQ (70 µM, 24 hr) treatment are shown from the total number of cut segments. Examples represent (i) short (1–2 mm) pre- and post-pharyngeal fragments, (ii) anterior (‘A’) blastema cut from trunk fragment exposed to PZQ (70 µM) for 1 day, (iii) posterior (‘P’) blastema cut from trunk fragment exposed to PZQ (70 µM) for 1 day, (iv) head fragment of sexualized D. japonica and (v) trunk fragment of sexualized D. japonica worm. (2.52 MB DOC) [file pntd.0000464.s004.doc]

**Supplementary Figure 2**

**
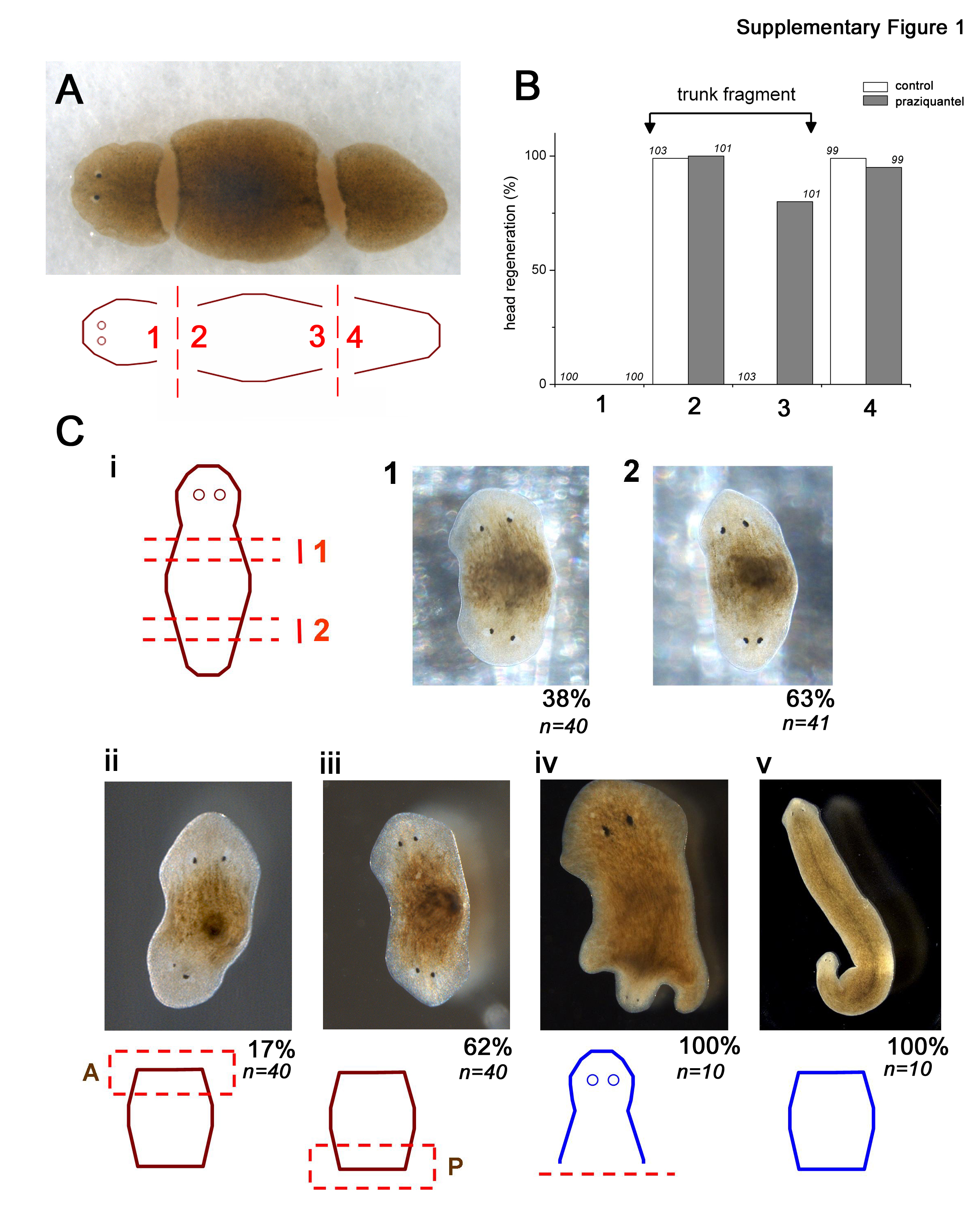
**

**Supplementary Figure 2**

**PZQ is efficacious at anteriorizing different types of planarian fragments.** (**A**) Image and schematic representation of basic trunk fragment assay, where two cuts (dashed) yield three fragments (‘head’, ‘trunk’ and ‘tail’ fragments) and four blastemas (‘1’-‘4’). (**B**) Results from the procedure shown in (A) scored as proportion of fragments regenerating heads from indicated blastema (‘1’-‘4’) in control (open bars) and PZQ-treated worms (solid bars, 70μM, 24hr). Number of fragments shown as italicized label from a cumulative dataset. (**C**) Gallery of bipolar worms produced by PZQ (70μM, 24hr) treatment from different cut fragments (cartoons) from asexual (i – iii, brown cartoon) and sexualized (iv & v, blue cartoon) worms. For each example, proportion of bipolar fragments by PZQ (70μM, 24hr) treatment are shown from the total number of cut segments. Examples represent (i) short (1-2mm) pre- and post-pharyngeal fragments, (ii) anterior (‘A’) blastema cut from trunk fragment exposed to PZQ (70μM) for 1 day, (iii) posterior (‘P’) blastema cut from trunk fragment exposed to PZQ (70μM) for 1 day, (iv) head fragment of sexualized *D. japonica* and (v) trunk fragment of sexualized *D. japonica* worm.
