# Supplementary material for: A Novel Biological Activity of Praziquantel Requiring Voltage-Operated Ca2+ Channel β Subunits: Subversion of Flatworm Regenerative Polarity
Source: PLoS Negl Trop Dis. 2009 Jun 23;3(6):e464. doi: 10.1371/journal.pntd.0000464 (PMC2694594; doi:10.1371/journal.pntd.0000464)
Supplement: Figure S3 — Characterization of D. japonica Cavβ1 and Cavβ2. (A) Alignment of D. japonica Cavβ1 (551 amino acids) and Cavβ2 subunits (652 amino acids). Identical residues are shown in yellow, similar residues in green. Both proteins show highest homology in their SH3 (blue) and guanylate kinase domains (red) as illustrated in the similarity projection in (B). Residues in the GK domain shown to wall the α-interacting domain pocket are highlighted (*, [35]).Residues previously implicated in determining PZQ sensitivity are shown (arrow, [10],[11]). (C) In situ hybridization of Cavβ1 (top) and Cavβ2 mRNA (bottom) in ventral view shown in intact worms (left) and regenerating worms (2 days post cutting, right). Staining in pharynx (green arrows) and brain (red arrows is shown). Cavβ2 staining occurs in the anterior and posterior of the pharynx region (*). (D) RT.PCR analyses of mRNA distribution in head (‘h’), trunk fragments (‘p’ for pharynx) and tail (‘t’) sections for Cavβ subunits, as well as loading controls (β-actin) and regional markers (‘opsin’, head-specific; ‘Hox9’ posteriorly-biased marker). Primers: actin: 5′-TGGGACGATATGGAGAAGATCTGGCAT-3′, 5′-GCATACGATCAGCAATACCAGGGTA-CA-3′; opsin: 5′-CACCGCCATTTTTTGGTTTGGAAA-3′, 5′-GCAAATAGCACTGGTAGTT-CAGCAG-3′; hox9: 5′-GATTCTGCCTTCGGTAAATCTGACAT-3′; 5′-GCAATTTCCCACG-TTTTTGTCTAGT-3′; Myosin: 5′-AACGACGAACTGAAATGCCACCTCA-3′; 5′-CAGCTTG-TTCTTCTCTGGGTCTTTGT-3′; Cavβ1: 5′-TTACAAGATGCATGTGAACATC-3′; 5′-TAA-ATGGAGAATGCGCTATATC-3′; Cavβ2: 5′-ATTCAACAAATAAAATAAAAACTC-3′, 5′-TCGATATCCCAATATTCTATCTGC-3′. (1.64 MB DOC) [file pntd.0000464.s005.doc]

**Supplementary Figure 3**


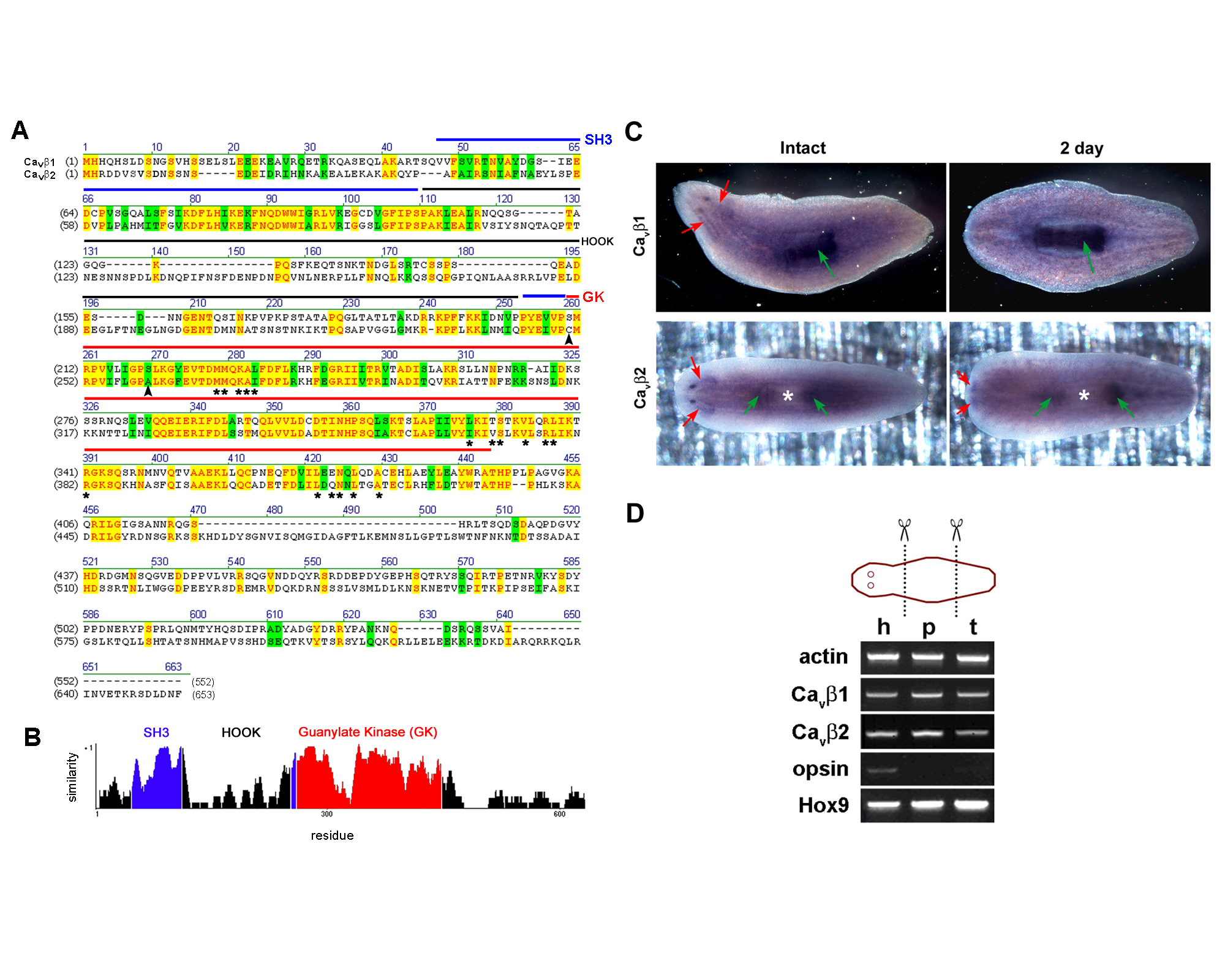


**Supplementary Figure 3**

**Characterization of *D. japonica* Ca_v_β1 and Ca_v_β2** (**A**) Alignment of *D. japonica* Ca_v_β1 (551 amino acids) and Ca_v_β2 subunits (652 amino acids). Identical residues are shown in yellow, similar residues in green. Both proteins show highest homology in their SH3 (blue) and guanylate kinase domains (red) as illustrated in the similarity projection in (**B**). Residues in the GK domain shown to wall the α-interacting domain pocket are highlighted (*, [35]).Residues previously implicated in determining PZQ sensitivity are shown (arrow, [10, 11]). (**C**) *In situ* hybridization of Ca_v_β1 (top) and Ca_v_β2 mRNA (bottom) in ventral view shown in intact worms (left) and regenerating worms (2 days post cutting, right). Staining in pharynx (green arrows) and brain (red arrows is shown). Ca_v_β2 staining occurs in the anterior and posterior of the pharynx region (*). (**D**) RT.PCR analyses of mRNA distribution in head (‘h’), trunk fragments (‘p’ for pharynx) and tail (‘t’) sections for Ca_v_β subunits, as well as loading controls (β-actin) and regional markers (‘opsin’, head-specific; ‘Hox9’ posteriorly-biased marker). Primers: *actin*: 5'-TGGGACGATATGGAGAAGATCTGGCAT-3’, 5'-GCATACGATCAGCAATACCAGGGTA-CA-3’; *opsin*: 5'-CACCGCCATTTTTTGGTTTGGAAA-3’, 5'-GCAAATAGCACTGGTAGTT-CAGCAG-3’; *hox9*: 5'-GATTCTGCCTTCGGTAAATCTGACAT-3’; 5'-GCAATTTCCCACG-TTTTTGTCTAGT-3’; *Myosin*: 5'-AACGACGAACTGAAATGCCACCTCA-3’; 5'-CAGCTTG-TTCTTCTCTGGGTCTTTGT-3’; Ca_v_β1: 5’-TTACAAGATGCATGTGAACATC-3’; 5’-TAA-ATGGAGAATGCGCTATATC-3’; Ca_v_β2: 5’-ATTCAACAAATAAAATAAAAACTC-3’, 5’-TCGATATCCCAATATTCTATCTGC-3’.
