# Supplementary material for: A Novel Biological Activity of Praziquantel Requiring Voltage-Operated Ca2+ Channel β Subunits: Subversion of Flatworm Regenerative Polarity
Source: PLoS Negl Trop Dis. 2009 Jun 23;3(6):e464. doi: 10.1371/journal.pntd.0000464 (PMC2694594; doi:10.1371/journal.pntd.0000464)
Supplement: Figure S4 — Effect of PC2 RNAi on worm mobility following sudden light exposure. Intact worms subject to PC2 RNAi (stained with red food color), and controls (stained green) were placed in a drop of water and video frames captured at 2 second intervals following exposure to white light. Stills show that PC2 RNAi worms (red) remain relatively immobile relative to the worms exhibiting the light aversion response (green). (0.64 MB DOC) [file pntd.0000464.s006.doc]

**Supplementary Figure 4**


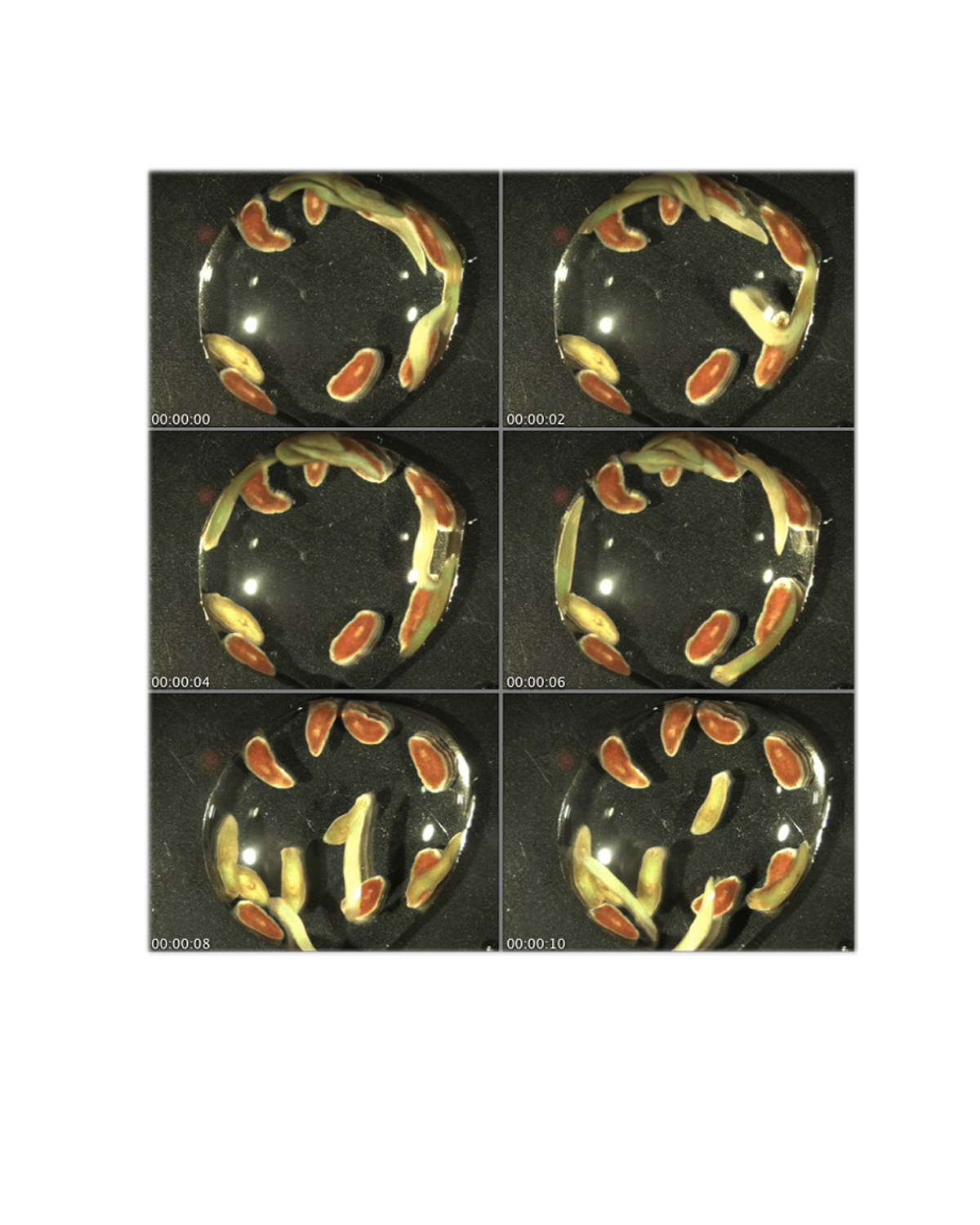


**Supplementary Figure 4.**

**Effect of *PC2* RNAi on worm mobility following sudden light exposure.** Intact worms subject to *PC2* RNAi (stained with red food color), and controls (stained green) were placed in a drop of water and video frames captured at 2 second intervals following exposure to white light. Stills show that *PC2* RNAi worms (red) remain relatively immobile relative to the worms exhibiting the light aversion response (green).
